# Analysis of public food procurement in relation to dairy products and their quality criteria

**DOI:** 10.3389/fnut.2023.1264389

**Published:** 2023-09-28

**Authors:** Katarzyna M. Brukało, Justyna Nowak, Neza Fras, Oskar Kowalski, Mojca Gabrijelčič Blenkuš

**Affiliations:** ^1^Faculty of Public Health in Bytom, Department of Health Policy, Medical University of Silesia, Katowice, Poland; ^2^Faculty of Public Health in Bytom, Department of Metabolic Disease Prevention, Medical University of Silesia, Katowice, Poland; ^3^Center for Analysis and Development of Health, National Institute for Public Health, Ljubljana, Slovenia; ^4^Faculty of Public Health in Bytom, Department of Human Nutrition, Medical University of Silesia, Katowice, Poland

**Keywords:** food policy, nutrition policy, public food procurement, school and kindergartens, dairy products, criteria

## Abstract

**Introduction:**

Public procurement of food is key to ensuring proper nutrition with high-quality products in public institutions such as schools and kindergartens. However, it should not be considered a mandatory expenditure from public finances but rather an investment in health promotion.

**Materials and methods:**

A total of 1,126 public procurement orders processed by schools and kindergartens in Poland during the period from November 2022 to March 2023 were analyzed. Ultimately, 197 public procurement orders meeting the inclusion criteria were considered for analysis. Based on these orders, 2,753 food products classified as dairy and its derivatives were extracted. The ordered quantities of individual products were analyzed, as well as their descriptions (quality characteristics).

**Results:**

Criteria related to composition were most commonly described, the most common criterion was the fat content and the absence of preservatives. On the second places were organoleptic characteristics, where taste and consistency expectations were most frequently specified. Sustainable public procurement criteria were the least frequently mentioned and were treated as highly marginal.

**Conclusion:**

Introducing minimum standards for the descriptions of dairy products in terms of organoleptic characteristics, composition features, and sustainability criteria will improve the quality of dairy products supplied to public institutions, particularly schools and kindergartens.

## Introduction

Mass nutrition of children in kindergartens and schools is a difficult issue that requires special attention. The main tool for its implementation are public procurements, thanks to which purchases of food products or full meals are carried out. On the one hand, the ordered products should be of the highest quality, on the other hand, the economic criterion is often a limitation. These issues become particularly important in terms of building eating habits among children and adolescents who spend most of the day at school and kindergarten.

Throughout childhood and early adolescence, nutrition plays a primary role in the growing, muscle and skeletal development, fat accumulation, as well as the risk of non-communicable diseases later in life. During the period of growth, both undernutrition and obesity have an impact on the maturation of multiple physiological systems (1). Long-term eating habits are established during childhood, a critical period that can influence the future risk of metabolic diseases, overweight or obesity, and other nutrition-related illnesses. The majority of children and adolescents have unsatisfactory or unhealthy diets. This is evident from the alarming trend of a 10-fold increase in the number of overweight and obese children worldwide over the past 40 years (2). According to data from the World Health Organization (WHO) in 2020, there were 39 million children under the age of 5 who were overweight or obese. Unfortunately, this problem also affects Poland. The WHO report indicated that overweight and obesity were reported in 32% of Polish children aged 7–9 years. It is the eighth place among the surveyed countries in Europe. According to the Polish Society for the Treatment of Obesity, in Poland, overweight or obesity occurs in: 12.2% of boys and 10% of girls in preschool children, and in 18.5% of boys and 14.3% of girls in school children (3). Preventing or improving many nutrition-related diseases can be achieved by changing lifestyles, particularly through adopting well-balanced diets. First, the family environment, and next, the school environment, play active and important roles in shaping the eating behaviors of young people. Given that nutrition is a cornerstone of investments in human capital, there is a need to scale up research aimed at promoting healthy eating in the school environment (2).

Public procurement of food in Poland is regulated by a law generally dedicated to public procurement (4). This means that there are no specific or targeted solutions dedicated to nutrition policy in these regulations. According to the position of the Public Procurement Office, the food procuring entity has the possibility to take into account a number of environmental factors (e.g., purchasing organic, seasonal, and sustainably produced food), but these are not obligatory, only suggestions (5).

On the other hand, Polish food law is based on both domestic regulations and those of the European Union. The primary legislative framework in this regard is the Food Safety and Nutrition Act of 2006 (current amended version from 2020) (6). It contains general provisions governing food law in Poland. However, it is important to note that the food market in Poland is diverse, including the quality of available products. Therefore, in the context of public procurement, it is not sufficient to merely specify a product’s general name (e.g., fruit yogurt); it is necessary to indicate its specific characteristics quality criteria (i.e., sugar or fat content, fruit content percentage).

To streamline and facilitate the public procurement process in Poland, the E-procurement Platform has been in operation since 2022. Each entity performing public procurement must log in and enter all relevant documents. An integral part of the E-Procurement Platform is the Public Procurement Bulletin, which allows you to search for and obtain information about a specific public procurement. In accordance with the rules of operation of the E-Procurement Platform, all documents related to a specific public procurement are deleted within 3 months of its completion.

According to the Education Law Act, kindergartens and primary schools in Poland are obliged to provide students with one hot meal during the day (in a voluntary and paid form) and the conditions for its consumption (7). In order to improve the solutions in place, the Minister of Health issued a regulation specifying the criteria for products allowed for sale in kindergartens and schools and the rules for composing the menu (8). However, they do not have a direct impact on the issue of public procurement of food by these units (they do not apply to ordered products).

One of the key product groups in baby nutrition is milk and dairy products. According to the Polish law, at least two portions of milk or dairy products should be served every day in educational units (8), while throughout the day it is recommended that children and adolescents should consume per day 3–4 servings of milk, yogurt, kefir, buttermilk, and partially with cheese (9). Dairy products are the best source of easily absorbable calcium, which is essential for building healthy bones and teeth. These products also contain complete proteins, as well as vitamins and minerals.

## Aim of the study

The aim of the study was to analyze the criteria used in the public food procurement procedure for schools and kindergartens. In Poland, no specific criteria are mandatory, which results in low-quality purchased products. However, some educational institutions choose to implement their own criteria to improve this quality. To this end, a quantitative and qualitative analysis of products from the milk group and its products was carried out and qualitative criteria in terms of product composition, organoleptic characteristics and sustainable public procurement were defined and verified.

## Materials and methods

In order to collect information on public tenders carried out by kindergartens and primary schools for food products ordered for 2023, data collection was carried out from Public Procurement Biulletin between 15 November 2022 and 15 March 2023.

In order to obtain information on tenders concerning the purchase of food products, the resources of the database were searched on the basis of CPV codes (15000000-8—Food, beverages, tobacco, and related products and all related products from the group of milk and milk products, a detailed list is included in Appendix 1).

On this basis, a database was created covering 1,126 items—public procurement, which in a given period were processed by the above-mentioned educational units in the scope of the indicated CPV codes.

The final criterion for inclusion in the analysis was the first procurement (those in which part of the procedure was repeated due to the invalidity of one of the parts was excluded) and the availability of full documentation to guarantee the reliability of the analysis carried out.

In the end, 197 public contracts were extracted, which together met the following criteria:

– have been processed by an educational unit (kindergarten, primary school, or school-preschool complex);
– were processed for the first time (covered the full commodity demand of individual units); and
– had complete documentation (description of the subject of the order, assortment and price form, draft provisions of the contract, information on the assumed amount allocated for the purchase of products, and the settlement of the procedure).

**Figure 1:**
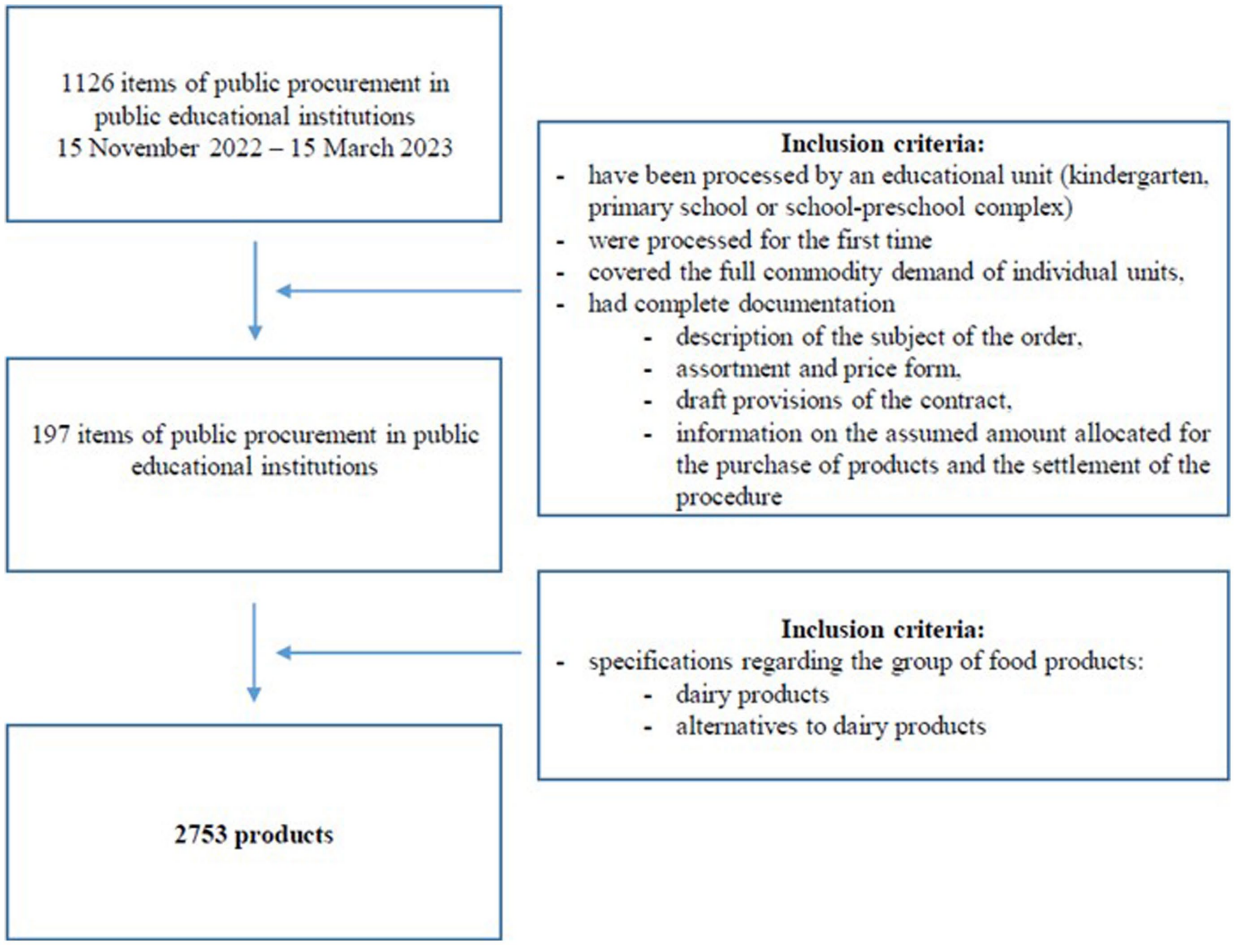
Characteristic of inclusion criteria.

Based on the collected documentation, a database of ordered dairy products and milk was created, which included 2,678 products belonging to this group (Figure 1).

According to some classifications, butter is also included in the group of milk and its preparations. Since this analysis focuses primarily on the quality of products and the volume of orders and their potential impact on children’s health, it was decided that butter would not be included here (it was classified as a fat group).

In the last step, a quantitative analysis was carried out for each product. In order to be able to compare the quantity of ordered products for estimation purposes, the following conversion factors were used:^1^

100 mL of natural yogurt – 103.6 mg,
100 mL of Greek yogurt – 118.3 mg,
100 mL buttermilk/kefir – 102 mg,
100 mL of cream – 104 mg.

Quantitative data were supplemented with a qualitative analysis—taking into account the criteria that are given by the Employers for individual food products classified in this group.

Based on the preliminary review of the collected descriptions of individual products, the following quality criteria were identified:

• in terms of product composition:

o fat content,
o protein content,
o sugar content,
o without added sugar,
o glucose-fructose syrup,
o without sweeteners,
o free from thickening and stabilizing substances,
o without preservatives,
o free from artificial colors, flavorings, and flavor enhancers,
o without milk powder,
o without genetically modified starch,
o non-GMO,
o free of edible gelatine, and
o a specific composition;

- in terms of organoleptic characteristics:

- o taste,
- o smell,
- o color, and
- o appearance/consistency;

• in the field of sustainable public procurement:

o locality of products,
o organic products,
o packing method (packing environment friendly), and
o packing volume.

To the best of the authors’ knowledge, this is the first detailed and comprehensive analysis of public procurement of a specific group of food products.

## Results

The group of milk and its products contained 2,678 products, which were divided into subgroups. The applicable breakdown is shown in the following table (Table 1).

**Table 1:** Breakdown of dairy products—volume, number of products and volume.

Type of dairy products	Number of products		Order volume	
	Number of products (*n*)	% of all products	Order volume (kg)	% of order volume
Milk	375	14.00%	550,496,75 (l)	70.22%
Yoghurts	612	22.85%	76800.17	9.80%
Cream	428	15.98%	51270.17	6.54%
Cheeses	611	22.82%	64,709,626	8.25%
Rennet cheese	494	18.45%	19564.76	2.50%
Kefir, buttermilk, curdled milk	158	5.90%	21121.1 (l)	2.69%
Grand Total	2,678	100.00%	783,962,576	100.00%

Detailed analysis of each of the groups is presented below.

### Milk

375 products were classified into the milk category, of which the largest group was cow’s milk. A detailed summary is presented in Table 2.

**Table 2:** Type and quantity of milk ordered for schools and kindergartens in 2023 (according to forecasts).

Type of milk	Number of products		Order volume	
	Number of products (*n*)	% of all products	Order volume (L)	% of order volume
Cow’s milk	245	65.33%	532130.4	96.70%
Vegetable milk	63	16.80%	6,068	1.10%
Lactose-free cow’s milk	27	7.20%	6491.5	1.18%
Flavored milk	27	7.20%	4811.4	0.87%
Flavored plant-based milk	6	1.60%	546.75	0.10%
Milk powder	3	0.80%	24.8	0.00%
Condensed milk	3	0.80%	33.65	0.01%
Goat’s milk	1	0.27%	200	0.04%
Grand total	375	100.00%	550496.75	100.00%

The most frequently (245 products, 65.33%) and the most numerous (532130.4 L, 96.70%) ordered milk was cow’s milk, while the least frequently milk powder (three products, 0.8%), concentrated (three products, 0.8%), and goat’s milk (one product, 0.27%).

The most preferred were UHT milk (108 products, 28.65%), then fresh (36 products, 9.55%), and pasteurized (33 products, 8.75%).

Milk with a fat content of 2% (147 products, 38.99%) or 3.2% (88 products, 23.34%) was selected. It should be emphasized that for 19 products (6.99%) the fat content has not been determined. In the case of 14 products from the cow’s milk category (5.71%), it was also determined that the protein content was equal to 3%.

In some facilities, lactose-free cow’s milk (27 products, 7.20%), vegetable milk (63 products, 16.80%), and goat’s milk (1 product, 0.27%) were also ordered as an alternative, which accounted for more than 25% of the ordered products.

Flavored and sweetened products were also not avoided—both in the case of cow’s milk (27 products, 7.20%) and in the case of plant alternatives (six products, 1.60%), which means that almost every 12th product was a flavored product.

In terms of composition, the Purchasers most often required that milk be free of antioxidants (27 products, 9.81%), without stabilizers (25 products) and without preservatives (21 products). In the case of vegetable milk, it was also required to be without added sugar (nine products) and without preservatives (eight products, 6.63%), as well as without dyes and flavor enhancers (eight products, 2.12%).

For organoleptic characteristics, guidelines on appearance, taste, smell, and consistency were given for 35 products and were mainly for cow’s milk (34 products, 11.37%). Most often, it was required that “the appearance and color be uniform, taste and smell clean without foreign flavors and smells, light cream color, liquid consistency.”

For 272 products (72.14%), no compositional criteria were set and for 341 products (90.45%), no organoleptic criteria were set. This means that in the case of 261 products (69.23%), the Purchaser did not define any quality criteria.

Milk in cardboard packaging (128 products, 34.13%) was preferred over in plastic bottles (33 products, 8.8%) or plastic bags (12 products, 3.2%). As a rule, these were packages with a capacity of 1 L, but it should be emphasized that in the case of 8 products (2.13%) the packaging volume was not given (which made it impossible to estimate the size of the order when the ordering party orders a certain number of pieces, but does not specify their size).

### Yoghurts

Another type of product was yogurt. They were divided into natural and flavored yoghurts and plant equivalents were distinguished (Table 3).

**Table 3:** Type and quantity of ordered yoghurts for schools and kindergartens in 2023 (according to forecasts).

Type of yogurt	Number of products		Order volume	
	Number of products (*n*)	% of all products	Order volume (kg)	% of order volume
Natural yogurt	314	51.31%	32703.18	42.58%
Natural plant-based yogurt	5	0.82%	128.00	0.17%
Flavored yogurt	285	46.57%	43740.49	56.95%
Plant-based flavored yogurt	8	1.31%	228.50	0.30%
Grand total	612	100%	76800.17	100%

The largest volume of ordered yoghurts included a group of natural yoghurts (51.31%). Of these, 44 items (14.01%) were Greek-style yoghurts.

Of the yoghurts, flavored yoghurts are the most frequently ordered (56.95%). 25 products of flavored yoghurts (8.77%, 2,835.18 kg) were described as probiotic yoghurts (according to the common advertising of a particular product). Every fifth yogurt (57 products, 20.00%) was a product in the form of a drink.

Only 30 products (10.53%) indicated that flavored yogurt should contain pieces of fruit, and 14 (4.91%) that the fruity aftertaste should be associated with the addition of fruit mousse. In the case of 14 products (4.91%), flavored yogurt was to be enriched with the addition of cereal flakes or cereal crisps (in chocolate).

Only in 98 cases has a maximum sugar content been established for flavored yoghurt. In 22 cases (7.72%) they were supposed to be completely sugar-free, in 40 cases (14.03%) below 10 g in 100 g of the finished product, in 16 cases (5.61%) below 13.5 g in 100 g of the finished product, and in 20 cases (7.01%) the maximum sugar content in flavored yogurts was set at 15/100 g of the finished product. The description of 15 products (5.26%) stipulated that the addition of glucose-fructose syrup is also excluded.

Educational units also ordered lactose-free products. More often they are natural yoghurts (13 products, 4.14% and one natural goat yoghurt—0.32%) than flavored yoghurts (five products, 1.75%). In addition, this offer was supplemented with plant substitutes (in accordance with Table 3).

Regarding the composition, the Purchasers most often expected that they would be yoghurts without the addition of: preservatives (96 products, 15.69%), food gelatine (70 products, 11.44%), stabilizers and thickeners (69 products, 11.27%), dyes (53 products, 8.66%), and milk powder (51 products, 8.33%).

In terms of organoleptic characteristics, yogurts were expected to have a dense texture (37 products, 6.05%), a mild and refreshing taste (33 products, 5.39%), a mild aroma (19 products, 3.1%), and a white and uniform color (17 products, 2.78%).

For natural (including vegetable) yoghurts, no compositional criteria were set for 195 products (61.13%) and no organoleptic criteria were set for 273 products (85.58%). In the case of 176 products (55.17%) from the group of natural yoghurts, no criteria were specified.

For flavored (including vegetable) yoghurts, no compositional criteria were defined for 162 products (55.29%) and no organoleptic criteria were defined for 275 (93.86%). 158 products (53.92%) did not have any criteria.

Among yoghurts, six products (0.98%) were ordered with the designation “bio,” and in the case of one product (1.63%) it was marked to be made from Polish milk.

Yogurts were packed mainly in plastic cups (53 products, 8.66%) or plastic bottles (24 products, 3.92%). For 28 products (4.58%), the weight or size of the ordered product was not even specified.

### Cream

428 products were qualified for the group of cream and cream. Coconut milk was also included in the analysis within this group, as an alternative to traditional cream and cream due to the similar fat content in the product (Table 4).

**Table 4:** Type and quantity of cream ordered for schools and kindergartens in 2023 (according to forecasts).

Type of cream	Number of products		Order volume	
	Number of products (*n*)	% of all products	Order volume (kg)	% of order volume
Cream	379	88.55%	47,798.77	93.23%
Cream	38	8.88%	3343.06	6.52%
Coconut milk	11	2.57%	128.34	0.25%
Grand total	428	100.00%	51270.17	100.00%

In this group, cream was ordered most often (379 products, 88.55%) and in the largest quantity (47,798.77 kg). In terms of fat content, cream dominated 18% (148 products, 39.5%), 12% (106 products, 27.97%), and 30% (84 products, 22.16%) It should be emphasized that for six products (1.58%) the desired fat content was not given. Sweet cream (52 products, 13.72%) was ordered more often than sour cream (33 products, 8.71%).

As an alternative to dairy products, they also ordered lactose-free cream (four products, 1.06%) and cream (one product, 2.63%) and, as a vegetable alternative—coconut milk (11 products, 2.57%). This means that 3.73% of all products in this category were lactose-free.

Products from this group were most often ordered as sterilized UHT method (76 products, 17.76%), homogenized (58 products, 13.55%), or pasteurized (15 products, 3.50%).

In terms of composition, they were expected to be free of preservatives (52 products, 12.15%) and thickeners such as genetically modified starch and carrageenan (eight products, 1.87%).

The taste of cream and similar/alternative products most often (48 products, 11.21%) was described as slightly sour or slightly sweet (depending on the type of cream) and mild and delicate, while the smell as pure (34 products, 7.94%). Consistency as uniform and creamy (44 products, 10.28%), and color also as uniform white with a light cream shade (55 products, 12.85%).

The Purchaser did not indicate any criteria in terms of organoleptic characteristics in relation to 367 products (85.75%), while in terms of composition—in the case of 371 products (86.68%). 282 products had no criteria (65.89%).

Creams were most often ordered in plastic cups (39 products, 9.11%) or cartons (57 products, 13.32%). In the case of 17 products (3.97%), the volume of the item or the size of the order was not specified. No sustainable procurement criteria have been identified.

### Cheese

The cheeses were divided according to the classification due to the production technologies.

The largest category was the category of acidic (curd) and curd-acidic (country cheeses) and acid-precipitation (homogenized cheeses; Table 5).

**Table 5:** Type and quantity of ordered acid, cottage cheese and acid and rennet cheeses for schools and kindergartens in 2023 (according to forecasts).

Type of cheese	Number of products		Order volume	
	Number of products (*n*)	% of all products	Order volume (kg)	% of order volume
Curd cheese	298	48.77%	42439.75	65.58%
Homogenized cheese	176	28.81%	18,644.756	28.81%
Curd sandwich cheese	90	14.73%	1,462.37	2.26%
Country cheese	30	4.91%	1225.5	1.89%
Goat curd cheese	7	1.46%	86.6	0.13%
Vegetable cheese	2	0.33%	2.9	0.00%
Flavored cottage cheese	4	0.65%	542	0.84%
Country flavored cheese	3	0.49%	303.5	0.47%
Smoked cheese	1	0.16%	2.25	0.00%
Grand Total	611	100.00%	64,709,626	100.00%

Of this group of products, the most frequently (298 products, 48.77%) and in the largest quantities (42439.75 kg, 65.58%) was ordered curd cheese. Purchasers most often chose semi-fat curd cheese (178 products, 59.73%), definitely less fat (17 products, 5.7%), or lean (six products, 2.01%). For 100 products, the fat content was not indicated (33.56%).

176 products (28.81%) were homogenized cheeses, of which 101 (57.39%) were flavored cheeses and nine were natural cheeses (5.11%). For the remaining 66 products (37.5%), the order does not specify whether it is a flavored or natural product.

Quite often, schools and kindergartens also order curd sandwich cheeses (fluffy, cream)—90 products (14.73%) in the amount of 1,462.37 kg (2.26%).

Also in this group, lactose-free products were ordered. These were 36 curd cheeses (12.08%), 24 homogenized cheeses (13.64%), and 10 curd sandwich cheeses (11.11%). In addition, seven goat curd cheeses (1.46%) and two vegetable cheeses (0.33%) were ordered.

In terms of composition, the Purchaser most often required that the products do not have added preservatives (75 products, 12.27%) and thickeners (22 products, 3.6%). In the case of homogenized cheeses, it was also noted that they were without the addition of: artificial flavors (16 products, 9.09%), pork gelatin (14 products, 7.95%) or artificial colors (14 products, 7.95%), stabilizers (12 products, 6.81%), glucose-fructose syrup (11 products, 6.25%), and milk powder (11 products, 6.25%).

The Purchaser expects the taste of curd cheese and derivative products to be delicate and typical, without foreign flavors (45 products, 7.36%), uniform color from white to slightly creamy (39 products, 6.38%), while the consistency is uniform and creamy (46 products, 7.53%).

No organoleptic criteria have been established for 559 products (91.49%) and no quality criteria have been established for 490 products (80.2%). This means that for 469 products (76.76%) no criteria were defined.

In the case of one product (0.16%), it was indicated that it should be made from Polish milk, and in the case of two products (0.33%), that they should be “bio.”

Preference was given to plastic packaging in the form of cups (29 products, 4.75%), and the descriptions of 34 products did not include the desired weight of the piece or the entire order (5.56%).

The second group of cheeses were rennet and other cheeses (Table 6).

**Table 6:** Type and number of rennet and others ordered for schools and kindergartens in 2023 (according to forecasts).

Type of cheese	Number of products		Order volume	
	Number of products (*n*)	% of all products	Order volume (kg)	% of order volume
Rennet hard cheese	288	58.30%	15719.17	80.34%
Mozzarella cheese	63	12.75%	1460.1	7.46%
Processed cheese	62	12.55%	989.83	5.06%
Feta cheese	60	12.15%	1053.03	5.38%
Mascarpone cheese	16	3.24%	290	1.48%
Mildew cheese	2	0.40%	8.25	0.04%
Semi-hard cheese	1	0.20%	30	0.15%
Haloumi cheese	1	0.20%	11.25	0.06%
Buratta cheese	1	0.20%	3,125	0.02%
Grand total	494	100.00%	19564.76	100.00%

The largest group among these products were hard rennet cheeses (288 products, 58.3%), which accounted for 4/5 of the order volume (15719.17 kg, 80.34%). Among them, the most popular were gouda cheese (101 products, 35.07%) and salami (20 products, 6.94%). In the case of 128 products (44.44%), its species was not precisely specified.

Also popular among the ordered products were mozzarella cheese (63 products, 12.75%) and feta cheese (60 products, 12.15%) and processed cheese (62 products, 12.55%). Units of education also ordered lactose-free rennet cheese (nine products, 1.82%).

The expected fat content was indicated for 53 products (10.72%) and ranged from 20 to 45% (for hard rennet cheese) and 10% for processed cheese.

In the case of 54 products (10.93%), it was indicated that they were preservative-free cheeses, and in the case of 15 products (3.04%) that they did not contain artificial dyes. For 12 products (2.43%), the specific expected composition was determined. No compositional criteria were set for 437 products (88.46%).

Taste requirements were taken into account for 67 products (13.56%), while the criteria for smell for 37 products (7.49%). In 60 (12.15%) descriptions, a description of consistency was included, and in 43 a description of the expected color (8.7%). No organoleptic requirements were included for 283 products (57.29%).

For 250 products (50.61%) in this category, no criteria have been defined regarding the composition or quality of the products.

No products that met the bio or eco criteria were also identified, and for 12 products (2.43%) no item size or order volume was given.

### Kefirs, buttermilk, and curdled milk

Kefirs, buttermilk, and settled milk were the least frequently ordered dairy products. In the list of ordered products, the milk sat only two times (190.25 kg), kefir appeared only 75 times (9312.03 kg), while buttermilk 89 times (12149.22 kg), of which in 14 cases it was flavored buttermilk (1294.38 kg).

In terms of composition, it was noted that these products were without the addition of preservatives (10 kefir, 13.33%; 11 buttermilk, 12.36%), thickeners (7 kefir, 9.33%; 9 buttermilk, 10.11%).

In terms of organoleptic characteristics, kefirs should be characterized by a slightly acidic, slightly sparkling and delicate taste (10 products, 13.34%), a uniform, dense, creamy consistency (seven products, 9.33%), and a mild and clean (no foreign odors) aroma (six products, 8.00%).

Buttermilk should have a mild or slightly sour taste (seven products, 7.87%), a clean smell (five products, 5.62%), and a uniform and dense consistency (five products, 5.62%).

No criteria (neither in terms of composition nor organoleptic characteristics) were defined for 63 buttermilk (70.79%) and 39 kefir (52.00%).

None of the products in this group has been described as bio or eco. In terms of packaging, buttermilk was most often ordered in cartons (19 products, 20.35%), while kefir—in plastic cups (four products, 5.33%).

### Collective sheet

Below, individual groups of dairy products are listed, including the criteria met.

Criteria related to composition were described most often, organoleptic characteristics less frequently, and sustainable public procurement criteria least often. In terms of composition, the most common criterion was fat content and no added preservatives. In terms of organoleptic characteristics, the expectations regarding taste and consistency were most often given, and the least often regarding the smell. Regarding the features of sustainable public procurement—they are treated completely marginally.

A detailed summary is presented in the Table 7.

**Table 7:** Summary of the number of dairy products for which a given feature is specified.

		Milk	Yoghurt	Curd cheese	Rennet cheese	Kefirs	Butter milks	Cream	TOTAL
Product composition	Fat content	260	119	218	53	14	13	411	1,088
	Protein content	14	21						35
	Sugar content	6	118	26					150
	Without added sugar	14	41	2		5			62
	Without glucose-fructose syrup		15	11					26
	Without sweeteners	7							7
	Free from thickening and stabilizing substances	25	69	22		7	9	8	140
	Without preservatives	21	96	75	54	10	11	52	319
	Free from artificial colors/flavorings/flavor enhancers	18	53/48/35	14/16	15				33
	Without milk powder		51	11					62
	Without genetically modified starch			8			3	8	19
	Non-GMO	7	4	8					19
	Free of edible gelatine		70	14					84
	A specific composition		47	40	12	9	5	x	113
Organoleptic characteristics	Taste	35	33	49	67	10	7	48	249
	Smell	35	19	3	37	6	5	34	139
	Color	35	17	39	43			55	189
	Appearance/consistency	35	37	46	60	7	5	44	234
Sustainable public procurement	Locality of products		1	1					2
	Organic products		6	2					8
	Packing method-cartonboard/glass	128					19	57	204
	Packing volume—no data	8	28	34	12			17	99
Total		648	792	609	353	68	77	734	

## Discussion

Well-balanced nutrition is one of the most important environmental factors that influence human development and the maintenance of good health. It involves meeting the body’s energy and essential nutrient requirements in their entirety (10).

Milk and dairy products are rich in many important nutrients like protein, calcium, magnesium, phosphorus, zinc, iodine, potassium, vitamin A, vitamin D, vitamin B12, and vitamin B2. Milk and other dairy products, like cheese, yogurt, and other fermented milks, provide the body energy, protein, micronutrients, and bioactive compounds that promote growth and development (11). Considering the above, it is crucial to ensure an adequate intake of these food products in the nutrition of children and adolescents.

In the nutrition of children in kindergarten and school facilities, there are many mistakes being made. One of them is insufficient intake of milk and dairy products. These observations are particularly concerning, especially among the young population (12).

Based on general Polish recommendations, children and adolescents should consume 3–4 servings of milk, yogurt, kefir, or buttermilk. Additionally, a smaller amount of cheese is recommended (10). Our results shown that the most volume ordered dairy products was milk (70.22%), yogurt (9.80%), and cheese (8.25%). On one hand, these results are quiet acceptable, but on the other hand, when we analyzed each type of product in this group, unfortunately, we observed a significant number of products containing sugar. Additionally, a significant proportion of cream (6.54%) was observed in the overall order, while the contribution of fermented dairy products such as kefir, buttermilk, and curdled milk was very low (2.69%).

Milk and dairy products provide over 50% of the total calcium intake in the average Polish diet (share of milk in the supply of calcium is around 21.5%). Milk and fermented dairy products contain approximately 118–120 mg of calcium per 100 g, curd cheeses range from 54 to 98 mg of calcium per 100 g, and rennet cheeses contain from 386 to 1,380 mg of calcium per 100 g (13, 14). According to the Dietary Guidelines for the Polish population, the recommended daily intake of calcium is 700 mg for children aged 1–3 years, 1,000 mg for children aged 4–9 years, and 1,300 mg for adolescents aged 10–18 years, which is the highest level among different age groups (15). Moreover, milk and fermented dairy products provides also other nutrients necessary for growth and development. Consumption of these kind of foods is certainly associated with health benefits and a reduced risk of many diseases (11, 13, 14, 16).

In light of this, it is crucial to ensure a consistent and high-quality supply of milk and dairy products in the daily meals served in educational institutions. The majority of the milk ordered was cow’s milk (96.7%), which is a positive outcome as it does not contain any additional additives such as sugar. However, among the yogurt options, the most popular choice was flavored yogurt (56.95%), while natural yogurt had a lower demand at 42.85%. This is concerning as flavored yogurt often contains added sugar and higher calorie content compared to natural yogurt. On a positive note, the most frequently ordered types of cheese were curd cheese and homogenized cheese (65.58 and 28.81% respectively), which is encouraging.

Generally, amount of rennet cheese in whole analyzed dairy products was the lowest—only 2.50% of order volume, but 18.45% of all products. In this subgroup the most order volume was rennet hard cheese (80.34%). Rennet cheese is rich in calcium, protein, and various minerals and vitamins. However, this product has a higher fat content, making it more calorie-dense. Polish recommendations suggest consuming rennet cheese in smaller amounts compared to milk and fermented milk products.

Also, our study revealed that educational units ordered a significant amount of cream (6.54% of the total order volume of dairy products). Cream is a high-fat milk product obtained by separating the fat from milk, and it is known for its high caloric content. In meal preparation, it is recommended to replace cream with fermented dairy products such as yogurt or kefir.

Not only the quantity but also the quality of the ordered products is important. It should be emphasized that some of the descriptions do not include such key data as the fat content (e.g., in relation to milk or cream) and the weight of the packaging. As shown in the analysis, almost half of them do not have any specific criteria—neither in terms of the quality of their composition nor in terms of organoleptic. On the one hand, it should be considered that some dairy products have specific minimum criteria to be approved for marketing or minimum guidelines, e.g., Polish Standard for milk and milk products. However, on the other hand, it should be taken into account that the quality of dairy products available on the Polish food market is very different, which will significantly affect the nutritional status of children and their nutritional behavior (17).

Among the criteria in terms of composition, apart from the fat content, the most frequently required is the lack of preservatives and stabilizing substances. Often, the customer also requires dairy products with a specific sugar content (which is not low, because on average it is 13.5 g/100 g of product). This type of action certainly does not correspond to the fight against overweight and obesity, so popular among children and adolescents.

In terms of organoleptic criteria, the Purchasers most often paid attention to taste and consistency.

In public procurement, changing nutritional trends can also be observed. It is worth noting the growing share of lactose-free dairy products and plant products. It may be worrying that the analysis identifies symbolic products that fall within the scope of sustainable public procurement.

It seems that in view of the growing nutritional awareness of society, sustainable food products will gain social acceptance (18), but it is necessary to provide tools to facilitate their definition, implementation and enforcement in public procurement (19). Good practice in this area and the roadmap may be the criteria for sustainable public procurement implemented by Finland (20), Slovenia (Slovenian (21)), Latvia (22), and Brazil (23).

Public procurement of food for educational units certainly requires reasonable and stable solutions (24–26). In the Member States of the European Union, they are not harmonized at Union level, but some of the Member States implement their specific solutions in a more or less detailed manner (27). Therefore, it is also necessary in Poland to take measures that will allow the promotion of minimum standards in the field of public food procurement in schools and kindergartens.

This analysis conducted regarding the most commonly used criteria (qualitative/related to product composition and sustainability) and the most frequently and most numerous ordered dairy products provides background for evidence-based policy-making and interventions. Based on the obtained results, policymakers can verify which criteria are most commonly used (and therefore most acceptable) and which types of dairy products are most popular among those purchased, and how this corresponds to dietary guidelines for children and adolescents. In the future, it would be valuable to expand this analysis to include a study of dietary habits among children and adolescents, as well as their nutritional status. This would be extremely helpful in defining the role of educational institutions in promoting proper nutrition among children and adolescents through the use of a tool—a catalog of criteria ensuring the high quality of public food procurements.

Based on the conducted analysis, a kind of catalog of criteria for use in public food procurement procedures has been created. Importantly, their implementation is feasible at the level of individual educational institutions, as well as at the level of local government or even as national solutions. These criteria can serve as inspiration for individuals involved in public procurement in schools and kindergartens, not only in Poland.

## Conclusion

Public food procurements for schools and kindergartens from an economic perspective are one of the main expenses, and from the perspective of children and adolescents, one of the main meals during the day. From a public health perspective, it should be an investment in health promotion. Nutritional products with a good composition will allow to prepare nutritious meals, and these will influence into the nutritional status of children and adolescents.

In situations where mandatory quality criteria are not specified, it leads to a reduction in the quality of purchased products. It is worth noting that dairy products are among the primary and most frequently consumed foods by children. Therefore, it is all the more important for the products consumed daily, multiple times, to be of the highest quality, and a tool for achieving this is the establishment of quality criteria in public procurements.

It is particularly important to establish and/or systematize standards for dairy products that children and adolescents consume at least two servings per day. As shown in the study, these standards should concern: composition, organoleptic features and sustainable public procurement. It is also important to increase the knowledge and competence of public procurements officers in the preparation of public procurement. This challenge often remains on the side of local governments, which play the role of the Purchaser.

## Data availability statement

The raw data supporting the conclusions of this article will be made available by the authors, without undue reservation.

## Author contributions

KB: Conceptualization, Data curation, Formal analysis, Investigation, Methodology, Project administration, Validation, Writing – original draft, Writing – review & editing. JN: Methodology, Writing – original draft, Writing – review & editing. NF: Writing – review & editing. OK: Supervision, Writing – review & editing, Conceptualization. MG: Project administration, Supervision, Writing – review & editing.

## Funding

The author(s) declare financial support was received for the research, authorship, and/or publication of this article. Project: Best Re-MaP, funded by the European Union’s Health Programme (2014-2020) Grant Agreement Number 951202.

## Conflict of interest

The authors declare that the research was conducted in the absence of any commercial or financial relationships that could be construed as a potential conflict of interest.

## Publisher’s note

All claims expressed in this article are solely those of the authors and do not necessarily represent those of their affiliated organizations, or those of the publisher, the editors and the reviewers. Any product that may be evaluated in this article, or claim that may be made by its manufacturer, is not guaranteed or endorsed by the publisher.

## Footnotes

^1^The conventer was developed on the basis of manufacturers’ indications on similar products.
