# Supplementary material for: Analysis of public food procurement in relation to dairy products and their quality criteria
Source: Front Nutr. 2023 Sep 28;10:1264389. doi: 10.3389/fnut.2023.1264389 (PMC10569494; doi:10.3389/fnut.2023.1264389)
Supplement: Supplementary file 1 [file Data_Sheet_1.docx]

Appendix No. 1

15000000-8 - Food, beverages, tobacco and related products

15500000-3 - Dairy products

15510000-6 - Milk and cream

15511000-3 - Milk

15511100-4 - Pasteurized milk

15511200-5 - Sterilized milk

15511210-8 - UHT milk

15511300-6 - Skimmed milk

15511400-7 - Semi-skimmed milk

15511500-8 - Whole milk

15511600-9 - Condensed milk

15511700-0 - Milk powder

15512000-0 - Cream

15512100-1 - Sour cream

15512200-2 - Fat cream

15512300-3 - Full-fat cream

15512900-9 - Whipped cream

15530000-2 - Butter

15540000-5 - Cheese products

15541000-2 - Table cheese

15542000-9 - Fresh cheese

15542100-0 - Curd cheese

15542200-1 - Soft cheese

15542300-2 - Feta cheese

15543000-6 - Compound cheeses, powdered, with mould overgrowth and other cheeses

15543100-7 - Cheese with mould overgrowth

15543200-8 - Cheddar cheese

15543300-9 - Combined cheese

15543400-0 - Parmesan cheese

15544000-3 - Hard cheese

15545000-0 - Cheese spreads

15550000-8 - Classified dairy products

15551000-5 - Yogurt and other fermented milk products

15552000-2 - Casein

15553000-9 - Lactose or lactose syrup

15554000-6 - Whey
